# *Chlamydia trachomatis* Polymorphic Membrane Proteins (Pmps) Form Functional Homomeric and Heteromeric Oligomers

**DOI:** 10.3389/fmicb.2021.709724

**Published:** 2021-07-19

**Authors:** Alison Favaroni, Johannes H. Hegemann

**Affiliations:** Institute of Functional Microbial Genomics, Heinrich-Heine-University, Duesseldorf, Germany

**Keywords:** *Chlamydia trachomatis*, polymorphic membrane proteins, adhesion, heteromers, oligomers

## Abstract

*Chlamydiae* are Gram-negative, obligate intracellular bacteria, which infect animals and humans. Adhesion to host cells, the first step in the infection process, is mediated by polymorphic membrane proteins (Pmps). Pmps constitute the largest chlamydial protein family, with 9 members (subdivided into six subtypes) in *C. trachomatis* and 21 in *C. pneumoniae*, and are characterized by the presence of multiple copies of GGA(I,L,V) and FxxN motifs. Motif-rich fragments of all nine *C. trachomatis* Pmps act as adhesins and are essential for infection. As autotransporters, most Pmp proteins are secreted through their β-barrel domain and localize on the surface of the chlamydial cell, where most of them are proteolytically processed. Classical autotransporters are monomeric proteins, which can function as toxins, proteases, lipases and monoadhesive adhesins. Here we show that selected recombinant *C. trachomatis* Pmp fragments form functional adhesion-competent multimers. They assemble into homomeric and heteromeric filaments, as revealed by non-denaturing gel electrophoresis, size-exclusion chromatography and electron microscopy. Heteromeric filaments reach 2 μm in length, significantly longer than homomeric structures. Filament formation was independent of the number of motifs present in the fragment(s) concerned and their relative affinity for host cells. Our functional studies demonstrated that only adhesion-competent oligomers were able to block a subsequent infection. Pre-loading of infectious chlamydial cells with adhesion-competent Pmp oligomers maintained the subsequent infection, while adhesion-incompetent structures reduced infectivity, presumably by blocking the function of endogenous Pmps. The very large number of possible heteromeric and homomeric Pmp complexes represents a novel mechanism to ensure stable adhesion and possibly host cell immune escape.

## Introduction

*Chlamydiae* are Gram-negative bacteria that replicate only within eukaryotic cells and can cause serious infections in humans and other animals (Elwell et al., 2016). *C. trachomatis* is the leading cause of sexually transmitted diseases and of preventable blindness in humans worldwide. Serovars A-C infect the ocular epithelium and recurrent infections may lead to trachoma formation (Ahmad and Patel, 2021). Serovars D-K target the urogenital tract and in 75% of young women the infection is asymptomatic. Left untreated, it can spread to the upper genital tract, inducing chronic inflammation and ultimately pelvic inflammatory disease and infertility (Murray and McKay, 2021). Despite the availability of antibiotic treatments, an effective vaccine is needed to control the infection rate (de la Maza et al., 2017, 2021). All species of *Chlamydiae* share a unique developmental cycle. Infection is initiated by the adhesion of the infectious elementary body (EB) to target cells. After internalization, the EB differentiates into the metabolically active reticulate body (RB) within a modified endosome known as the inclusion. The RBs are then able to replicate within the inclusion and eventually redifferentiate into infectious EBs, which leave the cell by lysis or extrusion to begin a new round of infection (Elwell et al., 2016; Christensen et al., 2019). Adhesion of the EB to the host cell is the first and essential step for the establishment of the infection and is a multifactorial process, mediated by an unknown number of adhesins (Hegemann and Moelleken, 2012).

In general, bacterial adhesins are categorized into two major classes, monomeric and filamentous. Fimbriae represent a group of filamentous adhesins. One example is found in the *E. coli* type 1 pilus, which is made up of the fimbrial subunit FimA and FimH serves as the adhesive tip (Kallas et al., 2020). Another example is the *E. coli* Curli protein, which is responsible for adhesion and biofilm formation. Curli is formed by the assembly on the cell surface of the secreted protein CsgA into long β-helical structures with amyloid properties (Barnhart and Chapman, 2006; Abdali et al., 2020). Non-fimbrial adhesins are short and are monomeric or trimeric. Due to their limited size, they allow a more intimate contact between the bacteria and the host cell (Berne et al., 2015). One of the major classes of non-fimbrial adhesins is the autotransporter (AT) family, of which *E. coli* AIDA-I is an example. The β-barrel of AIDA-I, which is inserted in the outer membrane (OM), exports the functional passenger domain (PD), which then is cleaved and functions as a membrane-associated adhesin following interaction with the β-barrel (Sherlock et al., 2004; Drobnak et al., 2015). In *Chlamydia*, only non-filamentous, monomeric adhesins have been described so far. In *C. trachomatis*, adhesins such as OmcB, Momp, Ctad1 and polymorphic membrane proteins (Pmps) are essential for infection (Moelleken and Hegemann, 2008; Liu et al., 2010; Becker and Hegemann, 2014; Stallmann and Hegemann, 2016).

Pmps form the largest protein family in *Chlamydiae* and are subdivided into six subtypes, with 9 members in *C. trachomatis* and 21 in *C. pneumoniae* (Grimwood and Stephens, 1999). Pmps are very heterogeneous not only in their number, but also in their sequence. The *pmp* genes are among the most variable regions in chlamydial genomes and have a high rate of mutation, not only among chlamydial species, but also among *C. trachomatis* serovars (Carlson et al., 2005; Holzer et al., 2020). While Pmps of the same subtype share significant amino acid sequence similarity, suggesting similar functions, different Pmps in different *Chlamydia* species share limited identity. For instance, the levels of overall identity within the entire Pmp families in *C. trachomatis* and *C. pneumoniae* are limited to 18 and 24%, respectively (Voigt et al., 2012; Van Lent et al., 2016; Holzer et al., 2020). Pmps are thought to act as ATs, with the PD being exposed to the extracellular space by the C-terminal β-barrel domain mediated by the β-barrel assembly machinery (BAM) (Henderson and Lam, 2001; Doyle and Bernstein, 2021). The PD is characterized by a high density of two peptide motifs, FxxN and GGA(I,L,V). Motif-containing segments of all *C. trachomatis* Pmps and of representative *C. pneumoniae* and *C. psittaci* Pmps act as adhesins, and are relevant for infection (Molleken et al., 2010; Becker and Hegemann, 2014; Favaroni et al., 2021). Pmps harbor multiple adhesion domains, but a minimum of two motifs can suffice for function (Molleken et al., 2010). Moreover, PmpD is shown to be involved in immune response modulation (Chu et al., 2020). For all these reasons, Pmps are considered valuable candidates for the development of a vaccine (Müller et al., 2017; Pal et al., 2017; de la Maza et al., 2021), therefore studying their biochemical and biological properties is of main interest. Proteome studies of *C. trachomatis* EBs and RBs revealed that most Pmps are processed during infection, generating fragments of different length and with different densities of the two repeat motifs (Saka et al., 2011). For *C. trachomatis* PmpD, various processed forms have been described during the life cycle and have been suggested as part of surface-associated protein complexes (Kiselev et al., 2009; Swanson et al., 2009). Bioinformatic analyses predict a triangular β-helical structure for Pmp PDs, which is typical for ATs and has been suggested to provide the basis for oligomerization (Hegemann and Moelleken, 2012). Indeed evidence for oligomerization of protein fragments of the PmpD subtype (*C. pneumoniae* Pmp21 and *C. trachomatis* PmpD) has been reported (Luczak et al., 2016; Paes et al., 2018).

In this study, we selected representative *C. trachomatis* A, D, G and I Pmp fragments bearing widely different numbers of motifs and with different length for investigation. We show for the first time that the analyzed Pmps interact with each other, forming different species of homomeric and heteromeric complexes, visualized as elongated protofibril-like structures. Filament formation is shown to be independent of the number of motifs, and of the level of their adhesive capacity. Functional studies revealed that only adhesion-competent oligomers were able to block a subsequent infection. Pre-coating of infectious chlamydial cells with adhesion-competent multimers was compatible with successful infection, while adhesion-incompetent structures reduced infectivity, presumably by blocking endogenous Pmp structures. Thus, this study presents evidence for the existence of functional, polyadhesive, polymeric autotransporter complexes.

## Materials and Methods

### Bacterial and Yeast Strains and Culture Conditions

The *E. coli* strains XL-1 blue (Stratagene) and Rosetta (Novagen) were used for plasmid amplification and protein expression, respectively. The *S. cerevisiae* strain CEN.PK2 was used for cloning of DNA fragments into plasmids. HEp-2 epithelial cells were grown at 37°C with 5% CO_2_ in Dulbecco’s Modified Eagle Medium (DMEM), supplemented with 10% heat inactivated fetal bovine serum, 1% non-essential amino acid solution, 1% MEM eagle vitamin mixture (Lonza), 2.5 μg/ml Amphotericin B and 50 μg/ml Gentamycin (Life technologies). *C. trachomatis* serovar E (DK-20) (London) (NCBI accession number: CP015304.1) was propagated in HEp-2 cells in cell culture medium supplemented with 12 μl/ml Cycloheximide (Sigma) and chlamydial EBs were purified by centrifugation through 30% Gastrografin (Schering), as previously described (Jantos et al., 1997).

### Plasmid Construction

Selected fragments of *pmpA* and *pmpG* were amplified from *C. trachomatis* serovars E genomic DNA and cloned into the expression vector pET24a (Novagen), fused to a C-terminal His-tag. Selected fragment of *pmpA* was amplified from *C. trachomatis* serovars E genomic DNA and cloned into the expression vector pAF14 (modified pET24a, by substituting the His-tag with a VSV-tag), fused to a C-terminal VSV-tag. All clonings were obtained by homologous recombination in *S. cerevisiae*, as previously described (Moelleken and Hegemann, 2008). *PmpD* and *PmpI* gene fragments were cloned into the expression vector pKM32, fused to an N-terminal His-tag, as previously described (Becker and Hegemann, 2014; Supplementary Table 1). The DNA sequence of all constructs was verified prior to use.

### Production of Recombinant Proteins

Protein expression was induced in *E. coli* Rosetta strain with 1 mM IPTG for 4 h. Cells were harvested by centrifugation at 5,000 rpm, cell pellets were lysed under denaturing conditions, using lysis buffer containing 6 M Guanidine/HCL overnight at 4°C, insoluble debris was removed by centrifugation at 24,000 rpm for 1 h. The soluble fractions containing His-tagged Pmps were loaded onto HiTrap chelating HP columns (GE Healthcare) and Pmps were specifically eluted in buffer containing 6 M Urea and 500 mM Imidazole. Pmps were renatured by dialysis against PBS at pH 7.4 (PmpD and PmpI) or pH 9 (PmpA and PmpG). Recombinant controls GST, CPn0473, and Ctad1 were produced as previously described (Fechtner et al., 2016; Stallmann and Hegemann, 2016).

### Immunoblotting and Coomassie-Staining

SDS-PAGE and immunoblot were performed as previously described (Sambrook and Maniatis, 1989). His- and VSV-tagged recombinant proteins and EBs were detected with monoclonal anti-His (Qiagen), anti-VSV or anti-Momp (Santa Cruz Biotechnology) antibodies, respectively, and visualized with AP-conjugated antibodies (Promega). Gels were stained with Coomassie Brilliant Blue G250 (Serva).

### Blue-Native PAGE and 2. Dimension SDS-PAGE

Blue Native-PAGE analyses were performed using the Native PAGE Novex 3–12% Bis-Tris Gel system, according to manufacturer’s protocol (Thermo Fisher Scientific) and analyzed by Coomassie staining. After Coomassie staining, selected bands were cut out, run into an SDS-PAGE and analyzed by immunoblot.

### Size Exclusion Chromatography (SEC)

SEC analyses were performed in PBS, on Superose6 10/300 GL, Superdex75 10/300 GL and Superdex200 10/300 GL columns (GE Healthcare), run at a flow rate of 0.3 ml/min, and 0.5 ml fractions were collected. The void volume (V_0_) was determined by using Blue dextran (2,000 kDa), and the separation range was defined by the elution of globular standard proteins, according to manufacturer’s protocols. Data were analyzed with PrimeView software (GE Healthcare).

### Pull-Down Interaction Assay

The VSV-tagged PmpA fragment was expressed in the *E. coli* strain Rosetta, cells were harvested as described above and lysed under native conditions with lysozim-containing buffer overnight at 4°C. Insoluble debris was removed by centrifugation at 24,000 rpm for 1 h at 4°C. The supernatant containing soluble VSV-tagged PmpA was then incubated with 1 mg/ml of refolded His-tagged protein overnight at 4°C and centrifuged at 12,000 rpm at 4°C. The soluble fraction was loaded on a HiTrap chelating HP column, His-tagged proteins were eluted with PBS containing 500 mM imidazole and analyzed by immunoblotting.

### Transmission Electron Microscopy (TEM)

Aliquots (10 μl) of rPmps (0.5 μM) were loaded onto glow-discharged S162 Formvar/carbon grids (Plano). Grids were washed with H_2_O, and the samples were negatively stained with 1% Uranyl acetate, and examined with an E902 electron microscope (Zeiss). Filaments characteristics were analyzed with ImageJ.

### Adhesion Assay

Soluble His-tagged recombinant proteins (100 μg/ml) were incubated for 1 h at 37°C with confluent monolayers of HEp-2 cells. Unbound proteins were removed by washing the cells three times with PBS. HEp-2 cells and bound proteins were solubilized with cell dissociation solution (Sigma), according to manufacturer’s protocol and analyzed by immunoblotting.

### Infection-Blocking Assays

In infection-blocking assays with soluble proteins, confluent monolayers of HEp-2 cells were incubated with 200 μg/ml recombinant proteins in cell culture medium for 2 h at 37°C. Unbound proteins were removed by washing the cells three times with PBS prior to infection with purified *C. trachomatis* EBs (MOI 10).

In the infection blocking assay with pre-coated EBs, purified *C. trachomatis* EBs were coated with 1 μM soluble proteins for 30 min on ice. Unbound proteins were removed by centrifugation at 15,000 rpm for 20 min at 4°C and coated EBs were used for infection of confluent monolayers of HEp-2 cells (MOI 10). Coating efficiency was checked by immunoblotting.

In both set-ups, the infection was performed for 2 h at 37°C without centrifugation in cell culture medium, supplemented with 12 μl/ml Cycloheximide. After 24 h, cells were fixed with 3% paraformaldehyde (PFA) and permeabilized with methanol. Anti-Momp (Santa Cruz Biotechnology) and Alexa-Fluor-488 anti-mouse (Invitrogen) antibodies were used for detection of chlamydial inclusions. Cells were visualized using a C2 confocal microscope (Nikon). Number of inclusions were counted with ImageJ.

### Statistical Analysis

The statistical tests used for each assay were calculated using GraphPad Prism 5 software. One-way ANOVA followed by Bonferroni’s multiple comparisons test was performed to evaluate the filament lengths in TEM analysis. In infection assays, One-way ANOVA followed by Dunnett’s multiple comparisons tests were performed to evaluate the relative infection rates.

## Results

### Pmp Proteins Form Homomeric High Molecular Weight (hMW) Complexes

**FIGURE 1:**
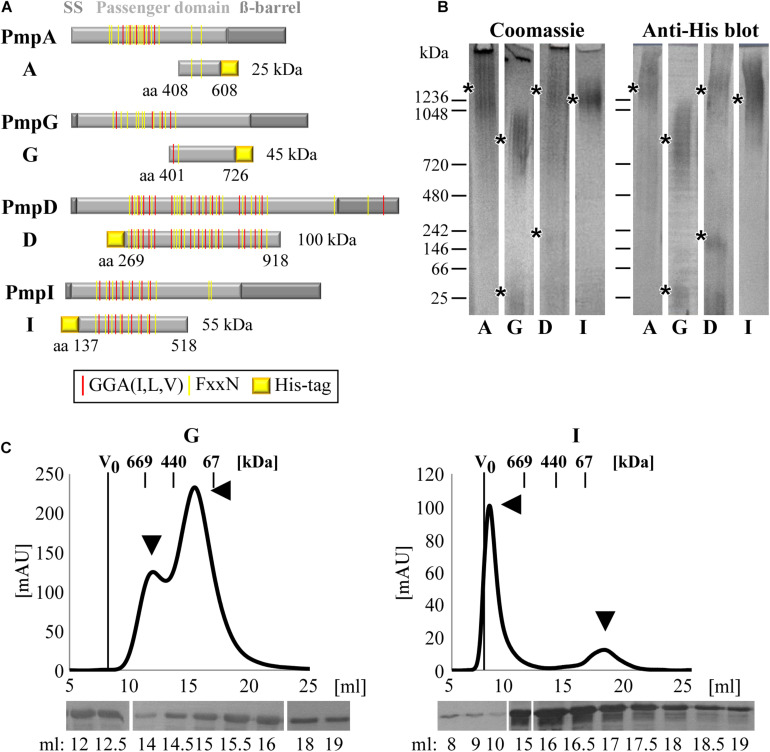
*C. trachomatis* Pmps form homomeric high-molecular-weight (hMW) complexes. **(A)** Schematic representation of *C. trachomatis* Pmps A, G, D, and I. Gray boxes indicate the characteristic autotransporter structure of Pmps, with the N-terminal signal sequence (SS), the central passenger domain (PD) and the C-terminal ß-barrel. Red and yellow lines in the PD indicate the GGA(I,L,V) and FxxN repeated motifs. The protein fragments analyzed in this study are indicated below the respective proteins with their amino acid positions and carry a His-tag. **(B)** Coomassie-stained and anti-His immunoblot of Blue Native-PAGE (BN) of renatured Pmp fragments (A, G, D, and I) (1 μg). Asterisks indicate the main bands. BN bands are cut for clarity purposes, original uncut pictures are shown in Supplementary Figure 6. Images are representative of three separate experiments. **(C)** Size exclusion chromatography (SEC) curves of motif-poor PmpG and motif-rich PmpI, obtained with Superose6 columns. The void volume (V_0_) is indicated. Elution volumes of globular standard proteins are indicated by short vertical lines. Arrowheads indicate relevant peaks. Anti-His immunoblots of relevant SEC fractions are shown at the bottom.

In order to investigate the molecular properties of Pmp proteins, representatives of four different subtypes from *C. trachomatis* serovars E were selected (Figure 1A). Fragments of PmpD (D^269–918^) and PmpI (I^137–518^) were selected as representative of motif-rich Pmp protein fragments and their functional abilities as relevant adhesins were previously shown (Becker and Hegemann, 2014). Fragments of PmpA (A^408–608^) and PmpG (G^401–726^) were selected as representative of motif-poor Pmp protein fragments, comparable to the motif-poor fragment analyzed for *C. pneumoniae* Pmp21 (Luczak et al., 2016). These proteins have been selected as representative for the different Pmp fragments, which have been predicted to be produced *in vivo*, harboring different numbers of motifs and representing different portions of the functional passenger domains (PD) (Saka et al., 2011). Segments of the PD of PmpD and PmpI, that are rich in repeated motifs, as well as fragments of PmpA and PmpG, which harbor only two motifs (motif-poor) each, were expressed as recombinant proteins. His-tagged motif-rich (D and I) and motif-poor (A and G) Pmp fragments were purified from *E. coli* and allowed to refold (Supplementary Figure 1).

The ability of each of these Pmp fragments to form oligomeric structures was then investigated by Blue Native-PAGE (BN), which separates proteins according to their size and conformation. All four Pmps migrated as broad heterodisperse bands centered at ∼1,300 kDa (A and D), ∼1,250 kDa (I) and ∼800 kDa (G), suggesting that each Pmp forms different hMW oligomeric species (Figure 1B). Only two Pmps (D and G) showed detectable amounts of dimers and monomers, respectively (low MW bands of 250 and 50 kDa), indicating preferential formation of hMW Pmp oligomers (Figure 1B). Further analysis of the composition of the different Pmp oligomers by Size Exclusion Chromatography (SEC) revealed that each Pmp exhibited a specific elution profile. SEC curves obtained with Superose6 columns show that motif-poor PmpG eluted in two peaks with apparent sizes of ∼650 and ∼200 kDa, and western blot analysis confirmed the presence of PmpG in all fractions (Figure 1C). Motif-rich PmpI eluted in a major peak of apparent size ∼2,000 kDa that partially overlapped with the void volume (V_0_). One smaller peak eluted at ∼60 kDa, representing the monomer. Western blot analysis confirmed the presence of PmpI in all fractions (Figure 1C). Motif-poor PmpA mainly eluted in a broad peak of ∼400 kDa and western blot analysis confirmed the presence of PmpA in all fractions (Supplementary Figure 2A). Finally, motif-rich PmpD eluted in two small hMW peaks of apparent sizes of ∼1,000 and ∼669 kDa, composed mainly of full-size PmpD fragment, as indicated by immunoblotting. Most of PmpD eluted in a peak centered at 140 kDa, which consisted mostly of degradation products (Supplementary Figure 2B).

Taken together, these data show that *C. trachomatis* Pmps have the ability to form different species of homomeric hMW complexes, independently of the number of motifs present.

### Different Pmps Interact With Each Other

**FIGURE 2:**
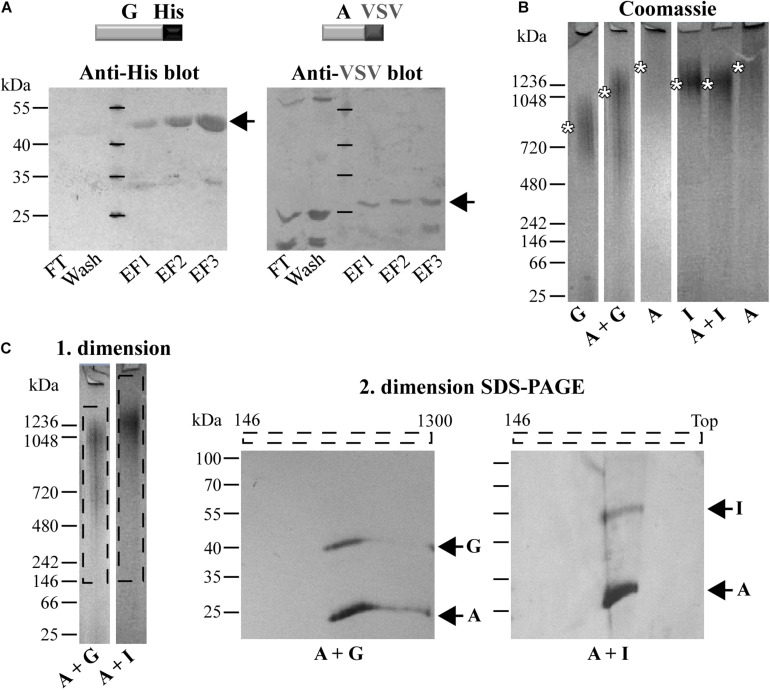
Different co-refolded Pmps form heteromeric hMW complexes. **(A)** Representative anti-His and anti-VSV immunoblots of pull-down assays of VSV-tagged PmpA (A-VSV) with His-tagged PmpG. FT, Flow Through; EF, Elution Fraction. **(B)** Representative coomassie-stained Blue Native-PAGE (BN) loaded with 1 μg of homomeric complexes (A, G, and I) and 1:1 co-refolded heteromeric complexes (A + G and A + I). Asterisks indicate the main bands. BN bands are cut for clarity purposes. **(C)** Representative 1. dimension BN bands containing the hMW A + G and A + I complexes (dashed box) analyzed by 2. dimension SDS-PAGE. Arrows indicate Pmp proteins. All images are representative of three separate experiments.

The ability of *C. trachomatis* Pmps to self-interact and form homomeric complexes led us to ask whether different Pmps might interact with each other. To systematically investigate the possibility that Pmps can mutually interact with each other, pull-down assays were performed. A VSV-tagged version of the motif-poor PmpA (A-VSV) was incubated with each of the other refolded His-tagged Pmp fragments. The protein solution was then affinity-purified using Ni-NTA columns, with affinity for the His-tag and subsequently examined for interaction via western blot (Supplementary Figure 3A). Control experiments confirmed the specificity of the system, as A-VSV was not able to elute in the same elution fractions as the His-tagged CPn0473, a *C. pneumoniae* adhesin (Fechtner et al., 2016), indicating a lack of interaction between the two proteins (Supplementary Figure 3B). Pull-down assays of A-VSV with His-tagged PmpG showed that the two motif-poor proteins eluted together in the same fractions, revealing a physical interaction (Figure 2A). Specific interactions were also shown for A-VSV with His-tagged motif-rich PmpD and PmpI, as PmpA was always eluted together with the His-tagged Pmps (Supplementary Figures 3C,D).

### Different Pmp Proteins Form Heteromeric hMW Complexes

Since Pmps have the ability to interact with each other, we investigated whether these interactions could result in the formation of hMW heteromeric complexes. This possibility was tested directly by analyzing refolded pairs of Pmps mixed in a 1:1 molar ratio by Blue Native-PAGE (BN). The presence of both fragments in the complexes was then tested by SDS-PAGE analysis of BN bands sections. Interestingly, the combination of motif-poor A and G Pmp fragments (A + G) gave rise to one broad band at ∼1,050 kDa, clearly distinct in size from either of the homomeric A and G Pmp complexes (Figure 2B). Co-refolding of Pmps A and I (A + I) yielded one main band of ∼1,250 kDa, comparable to homomeric I, but distinct from homomeric A (Figure 2B). The two protein pairs (A-G and A-I) were detected in the respective oligomers, when the 1. dimension BN bands of A + G and A + I Pmp complexes were analyzed by 2. dimension SDS-PAGE, albeit with different intensities: PmpA was more prominent than G and I, respectively (Figure 2C).

Similarly, co-refolded Pmps D and I (D + I) migrated as a hMW band centered at ∼1,250 kDa, comparable to homomeric I, but clearly dissimilar from homomeric D (Supplementary Figure 3E). Co-refolded Pmps A and D (A + D) showed one main hMW band at ∼1,050 kDa, again distinct from both homomeric A and D (Supplementary Figure 3E). Both proteins D and I were detected in the hMW band formed by the D + I complex, but I was more prominent than D. Similarly, A and D were both detected in the same region of the A + D BN band (Supplementary Figure 3F). These data suggest that two different Pmps can form heteromeric hMW complexes in which the stoichiometry of the different Pmps differs from the initial 1:1 ratio.

**FIGURE 3:**
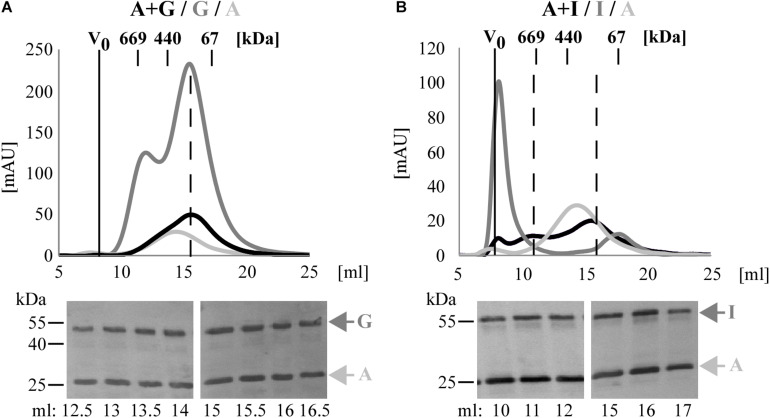
*C. trachomatis* Pmps form heteromeric hMW Pmp complexes. Superimposition of SEC curves of co-refolded A + G **(A)** and A + I **(B)** Pmp oligomers and their respective homomeric complexes, obtained with Superose6 columns. Homomeric complexes are represented in shades of gray and heteromeric complexes in black. The void volume (V_0_) is indicated. Elution volumes of globular standard proteins are indicated by short vertical lines. Vertical dashed black lines indicate peaks of the heteromeric Pmp complexes. Anti-His immunoblots of relevant SEC fractions are shown at the bottom. Arrows indicate Pmp fragments.

To confirm these data, the Pmp complexes A + G and A + I were further analyzed by SEC and their elution profiles were compared with those of the respective homomeric complexes. SEC curve of Pmp complex A + G, obtained with Superose6 column, eluted in a main broad peak of ∼200 kDa, overlapping with homomeric G, but clearly shifted from homomeric A. Western blot analysis confirmed the presence of both A and G Pmp fragments in similar amounts in all elution fractions (Figure 3A). SEC curve of Pmp complex A + I, obtained with Superose6 column, eluted in two peaks; one at ∼670 kDa, showing partial overlap with homomeric A but no overlap with homomeric I. In addition, A + I eluted in a peak of ∼200 kDa, partially overlapping with homomeric A but not with the I peak. Pmps A and I proteins were found in all elution fractions analyzed by immunoblotting (Figure 3B).

Taken together, these data show that heteromeric hMW complexes are formed when different denatured Pmps are mixed and allowed to refold together. Comparison of these complexes with the corresponding homomeric complexes by BN and SEC indicates that the heteromeric oligomers exhibit characteristics that clearly differentiate them from the respective homomeric complexes.

### Homomeric and Heteromeric Pmp Complexes Form Protofibril-Like Structures *in vitro*

**FIGURE 4:**
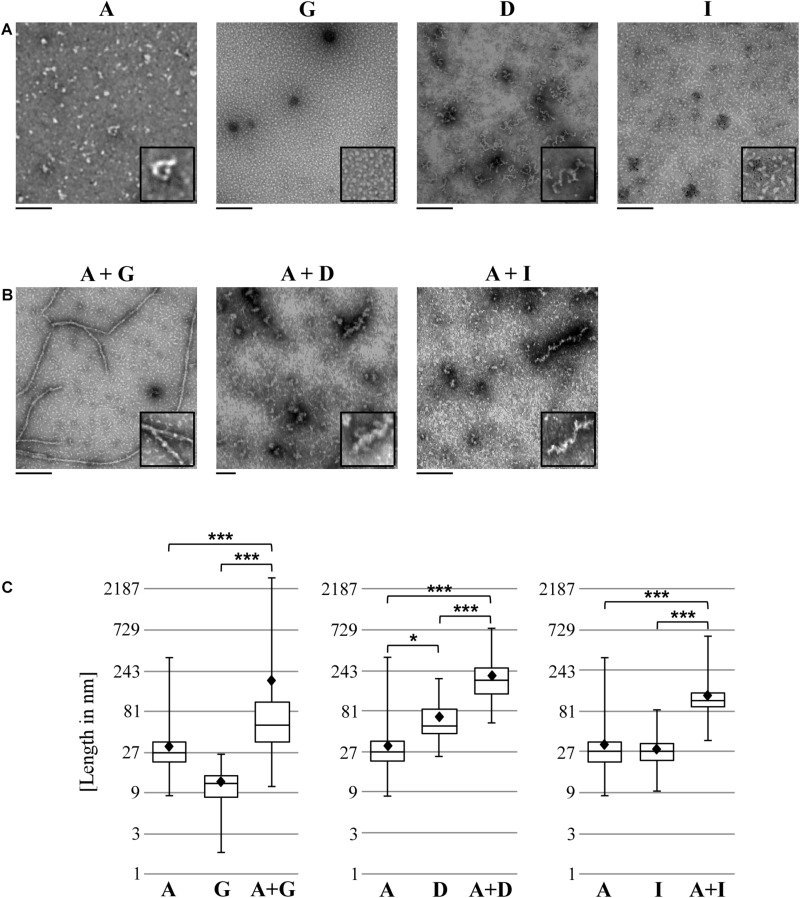
Heteromeric Pmp complexes form longer filaments than homomeric Pmp complexes. Transmission electron microscopy (TEM) pictures of 10 μl of 0.5 μM homomeric **(A)** and heteromeric **(B)** Pmp oligomers, representative of two (A, G, D, I, and A + G) and three (A + D and A + I) separate experiments. Two-fold enlargements are shown at the bottom right of each image. Scale bar: 250 nm. **(C)** Lengths of a total of 300 oligomers for each Pmp complex were measured with ImageJ and are displayed in boxplots on a logarithmic scale (base 3). Each heteromeric oligomer is compared to the respective homomeric oligomers. *P*-values were calculated on the means (shown as black diamonds) using One-way ANOVA followed by Bonferroni’s multiple comparisons test. *p < 0.05, ***p < 0.001.

We next examined the various homomeric and heteromeric Pmp oligomers by transmission electron microscopy (TEM). Pmp complexes were prepared using the same procedures used for Blue Native-PAGE and SEC analysis. For homomeric complexes, Pmp fragments were purified under denatured conditions and allowed to refold; for heteromeric complexes, pairs of Pmps in a 1:1 molar ratio were allowed to refold together. Intriguingly, all Pmp complexes exhibited different structures. Homomeric motif-poor G oligomers formed disk-like structures with an average diameter of 12 nm, while homomeric motif-poor A and motif-rich D and I were visualized as elongated protofibril-like filaments with average lengths of 32, 70, and 29 nm, respectively (Figure 4A). Surprisingly, heteromeric motif-poor A + G oligomers formed highly elongated structures of up to 2.9 μm in length, with an average length of 186 nm. Heteromeric A + D and A + I filaments had average lengths of 207 and 120 nm, respectively (Figure 4B). Interestingly, all heteromeric protofibril-like structures formed by A + G, A + I and A + D were significantly longer than the respective homomeric oligomers (Figure 4C).

These results support the idea that Pmps organize themselves into heteromeric oligomers, which differ markedly from their respective homomeric oligomers.

### Homomeric and Heteromeric Pmp Oligomers Show Differential Binding to Human Cells

**FIGURE 5:**
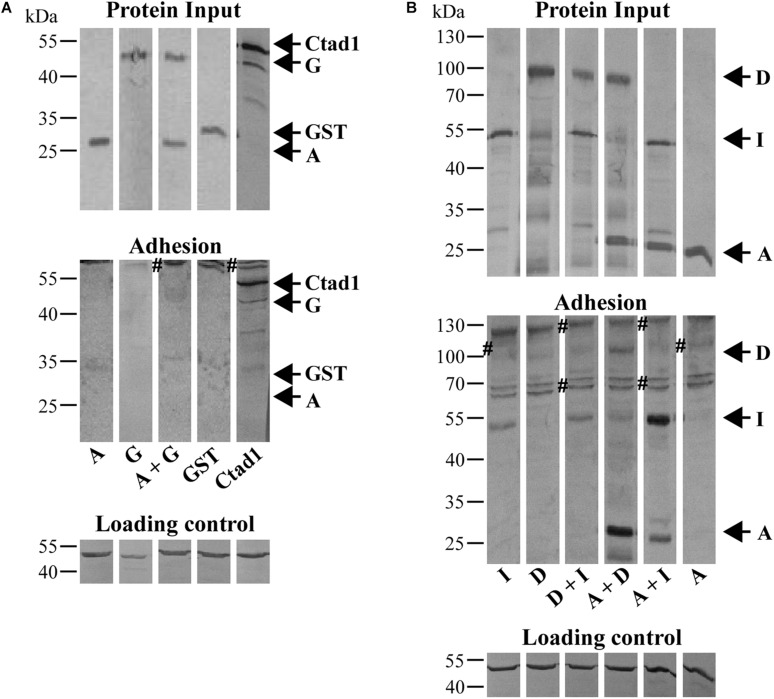
Cell-binding affinities of homomeric and heteromeric Pmp oligomers. **(A)** Binding affinities for HEp-2 cells of homomeric A and G, and heteromeric A + G Pmp oligomers (100 μg/ml). rGST and rCtad1 were used as negative and positive controls, respectively. **(B)** Binding affinities for HEp-2 cells of 100 μg/ml homomeric A, D, and I, and heteromeric D + I, A + D, and A + I Pmp oligomers. Anti-actin was used to verify the amount of HEp-2 cells (Loading control), anti-His antibody to determine the total input of recombinant proteins (Protein input) and their binding ability (Adhesion). Symbol “#” indicates non-specific bands. Arrows mark the positions of the Pmps analyzed. Western blots bands are cut for clarity purposes. Representative original uncut pictures of **(B)** are shown in Supplementary Figure 7. **(A,B)** All images are representative of three separate experiments.

Motif-rich fragments of all *C. trachomatis* Pmps have been characterized as adhesins that are relevant for infection (Becker and Hegemann, 2014). Therefore, we tested whether homomeric and heteromeric Pmp oligomers are functional structures, using a soluble binding assay. Soluble recombinant Pmp oligomers were incubated with human epithelial HEp-2 cells and the binding fractions were analyzed via immunoblotting. Recombinant His-tagged Ctad1, a *C. trachomatis* adhesin, and recombinant His-tagged GST were used as positive and negative controls, respectively (Stallmann and Hegemann, 2016; Figure 5A). Homomeric motif-poor A segment showed inconsistent binding and motif-poor G segment displayed no or very weak adhesion. Likewise, heteromeric motif-poor A + G oligomers displayed no binding ability (Figure 5A). The known homomeric, motif-rich adhesins Pmps D and I bound to the cells, although they showed a moderate binding in this set-up. Heteromeric motif-rich D + I was detected in the binding fraction, as were the respective homomeric Pmp oligomers (Figure 5B). Interestingly, the otherwise adhesion-inconsistent, motif-poor PmpA was always found in the binding fraction when presented as part of a heteromeric oligomeric complex together with the adhesion-competent motif-rich D or I Pmp fragment (Figure 5B).

Thus, homomeric and heteromeric Pmp oligomers differ in their binding affinity for human epithelial cells. The presence of the adhesion-inconsistent motif-poor PmpA in adhesion-competent, heteromeric complexes consisting of A and D or A and I suggests that otherwise non-binding Pmps can form part of adhesion-competent heteromeric oligomers.

### Adhesion-Competent Homomeric and Heteromeric Pmp Oligomers Are Relevant for the Infection

**FIGURE 6:**
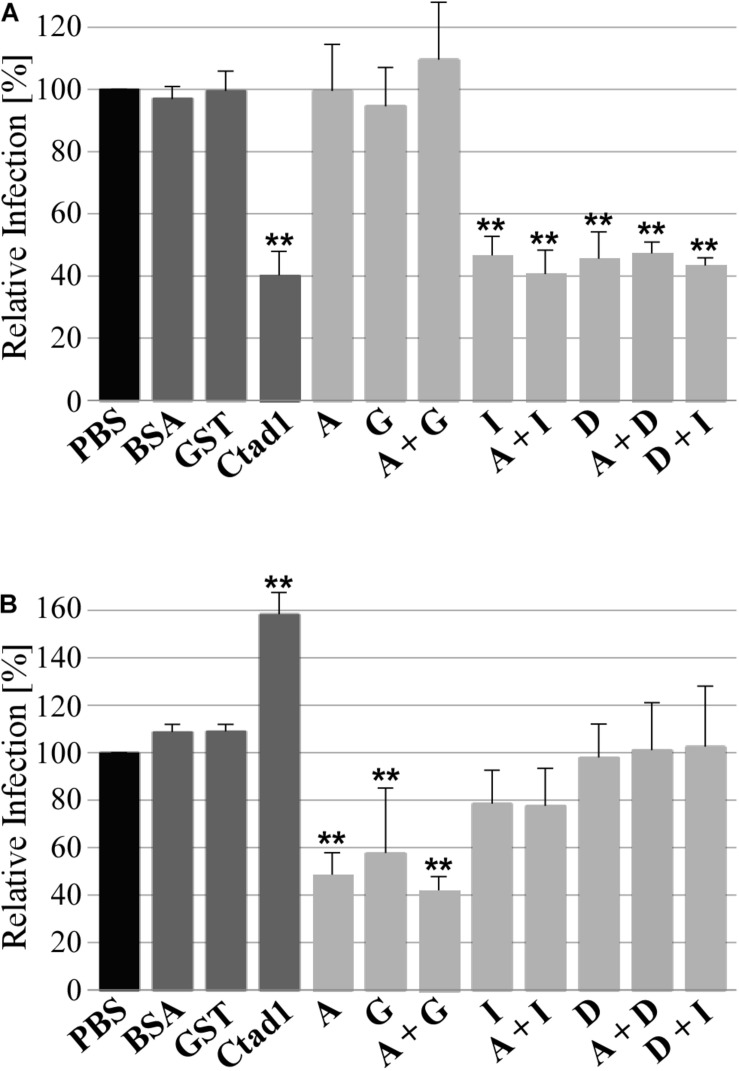
Adhesive homomeric and heteromeric Pmp oligomers are essential for infection. **(A)** Infection-blocking ability of 200 μg/ml soluble recombinant homomeric or heteromeric Pmp oligomers (light gray) incubated with human epithelial HEp-2 cells prior to infection with *C. trachomatis* EBs (MOI 10). **(B)** Infectivity of *C. trachomatis* EBs pre-coated with 1 μM soluble recombinant homomeric or heteromeric Pmp oligomers (light gray) prior to infection of human epithelial HEp-2 cells. BSA, rGST, and rCtad1 were used as controls (dark gray). Infection rates for each condition were determined by counting the number of inclusions in 10 pictures, for a total of 5.8 * 10^3^ HEp-2 cells and are expressed as a percentage of the number of inclusions determined by the PBS-treated sample (black) set to 100%. The relative infection rates represent the mean of three separate experiments. *P*-values were calculated using One-way ANOVA followed by Dunnett’s multiple comparisons test. **p < 0.01.

Finally, we tested the functional role of homomeric and heteromeric Pmp oligomers during infection of human epithelial HEp-2 cells by *C. trachomatis* in two infection blocking assays. In the first set-up, Pmp oligomers were incubated with epithelial cells prior to *C. trachomatis* infection. At 24 h post infection (hpi), the numbers of inclusions were quantified. If the recombinant protein complexes are able to bind to relevant receptors, they would block one infection route used by *C. trachomatis* EBs, thus this will reduce the infection rate. Pre-incubation of human cells with adhesion-competent recombinant homomeric (D or I) or heteromeric (D + I, A + D, or A + I) Pmp complexes consistently reduced *C. trachomatis* infectivity by approx. 60%, similar to the positive control rCtad1. Interestingly, inhibition by the recombinant heteromeric D + I oligomer was no more efficient than either of the homomeric oligomers. In contrast, adhesion-incosistent homomeric A, adhesion-incompetent homomeric G and adhesion-incompetent heteromeric A + G had no influence on *C. trachomatis* infectivity, similar to the negative controls BSA and rGST (Figure 6A and Supplementary Figure 4).

To confirm these results, the experimental set-up described above was reversed, and *C. trachomatis* EBs were pre-incubated with homomeric or heteromeric Pmp oligomers or with control proteins prior to infection of HEp-2 cells. In this set-up, we expect that the recombinant protein complexes would be able to bind to the endogenous Pmps or other adhesins present on the surface of the EBs. If adhesion-competent Pmp oligomers are binding to endogenous Pmps on the EB surface, they would substitute the function of the naturally expressed Pmps and mediate infection to the host cell. On the other hand, if adhesion-incompetent Pmp oligomers are binding to endogenous Pmps on the EB surface, they could mask the function of the naturally expressed Pmps and/or mask other adhesion-relevant chlamydial structures. In this scenario, a physiological interaction between *Chlamydia* and host cells would be prevented, resulting in a lower infectious rate. All Pmp oligomers tested (and the control proteins) were able to bind to the EB surface (Supplementary Figure 5A). EBs coated with adhesion-incompetent A, G and A + G oligomers reduced the infection rate by approximately 60%, compared to the PBS-treated sample, suggesting that they might bind and mask physiologically competent structures on EBs. In contrast, EBs coated with adhesion-competent D, I, D + I, A + D, and A + I Pmp oligomers had no significant impact on the infection rate, suggesting that adhesion-competent Pmp oligomers can bind to EBs and functionally replace the naturally exposed adhesive structures. The rGST control protein also bound to EBs, but did not alter their infectivity, indicating that its presence on EBs did not interfere with adhesion- or invasion-relevant structures. The positive control adhesin rCtad1 coated the EBs, possibly binding to itself and thus boosting the infection rate significantly (Figure 6B and Supplementary Figure 5).

These data together indicate that adhesion-competent homomeric and heteromeric Pmp oligomers are important for *C. trachomatis* infection, possibly acting on the same receptor pathway. Moreover, adhesion-incompetent fragments may contribute to the formation of adhesive heteromeric oligomers, supporting the adhesive-competent Pmps in their biological function.

## Discussion

*C. trachomatis* is an obligate intracellular human pathogen and must adhere to its target cells to establish an infection (Elwell et al., 2016; Murray and McKay, 2021). The nine Pmp adhesins represent the largest protein family in *C. trachomatis* and all are essential for infection (Becker and Hegemann, 2014). Most Pmps present in the infectious EB harbor one or more cleavage sites and indeed, processed forms of PmpD from *C. trachomatis* and *C. pneumoniae* have been detected, along with the full-length passenger domain (Wehrl et al., 2004; Swanson et al., 2009; Saka et al., 2011). In this study we selected segments of four different Pmp subtypes representing motif-poor (PmpA and PmpG) and motif-rich (PmpD and PmpI) fragments. Motif-rich PmpD and PmpI fragments were already shown to be adhesins, relevant for *C. trachomatis* infection (Becker and Hegemann, 2014). Motif-poor fragments of PmpA and PmpG are comparable to the motif-poor fragment of *C. pneumoniae* Pmp21 analyzed by Luczak et al. (2016). All the Pmp fragments analyzed in this study were able to elicit an immune protection against a *C. muridarum* challenge in mice (Pal et al., 2017). According to the cleavage sites identified by a proteome analysis of EBs and RBs, numerous *C. trachomatis* Pmp fragments, containing high and low densities of the two repeat motifs FxxN and GGA(I,L,V) may be produced *in vivo* (Saka et al., 2011), suggesting that different Pmp fragments might contribute to the infection in different ways during the developmental cycle.

Here, we show that motif-poor (PmpA and PmpG) and motif-rich (PmpD and PmpI) *C. trachomatis* Pmp fragments have the ability to interact strongly with each other, forming homomeric and heteromeric hMW complexes, as evidenced by pull-down, two-dimensional Blue Native/SDS-PAGE and SEC analyses. Interestingly, homomeric oligomer formation was also shown by Paes et al. (2018) for a 65 kDa fragment of PmpD from a different *C. trachomatis* serovar. Moreover, our electron microscopy analyses revealed that *in vitro* the homomeric complexes analyzed are able to form small, elongated filaments, while heteromeric complexes are comprised of significantly longer filaments, up to 2 μm in length. Importantly, adhesive affinity of homo- and heteromeric Pmp complexes is correlated with relevance for the *C. trachomatis* infection. Our study shows that although motif-poor Pmp A and G fragments harboring only two motifs each, form homomeric and heteromeric filaments, these structures have inconsistent or very little adhesion capacity. Thus, the ability to form homo- and heteromeric complexes is not linked to the number of repeat motifs present in the individual protein subunits. This contrasts with data for *C. pneumoniae* Pmp21 that show that a Pmp21 fragment requires only two motifs for oligomerization, adhesion and infection-blocking capacity (Molleken et al., 2010; Luczak et al., 2016).

In our study, TEM analyses show that Pmps under *in vitro* conditions form complexes with specific characteristics, organizing themselves into elongated protofibril-like structures, with heteromeric Pmp oligomers being significantly longer than homomeric oligomers, suggesting that heteromerization is favored. The lengths of these filaments are independent of the numbers of motifs in the individual Pmps tested. Similar filamentous structures have been observed for homomeric *C. pneumoniae* Pmp21, and shown to exhibit amyloid characteristics. Interestingly, the homomeric oligomers of Pmp21 fragment were able to bind to the host cell and influence the *C. pneumoniae* infection more efficiently than the Pmp21 monomers (Luczak et al., 2016). Taken together, all these data suggest a functional significance of homomeric and heteromeric Pmp oligomers for the early infection process. For the first time, we provide evidence that bacterial autotransporter proteins have the ability to form homomeric and heteromeric high molecular weight complexes with adhesion and infection-blocking capacities.

In general, all nine *C. trachomatis* Pmps are expressed and have been detected in proteome studies (Tan et al., 2010). Interestingly, in infectious EBs seven Pmps have been detected so far, and for six of them, beside the full-length proteins, proteolytically processed forms were predicted, harboring different numbers of the two peptide motifs and with different length (Kiselev et al., 2009; Saka et al., 2011). It is unclear whether the processed passenger domains (PD) remain tethered to the outer membrane (OM) via the translocator domain or are released into the extracellular environment. The presence of the *in vivo* processed forms of Pmps on the surface of the EBs has not yet been confirmed. However, the Pmp fragments analyzed in this study may be considered as representative of naturally generated fragments, and therefore serve as proof-of-principle for the abilities that this family of proteins harbors. Interestingly, post-translational processing of chlamydial autotransporters seems to be developmentally regulated, as it has been shown for PmpD. During the infection cycle, different processed forms of PmpD are generated, and are part of soluble and insoluble complexes of undefined composition, indicating that the different fragments may have different functions (Kiselev et al., 2009; Swanson et al., 2009). Function and mode of action of processed Pmps *in vivo* are not yet understood. Notably, however, in *C. pneumoniae* Pmp21, multiple adhesive domains have been identified within the PD, indicating that one function of unprocessed and processed Pmp21 forms is adhesion-related (Molleken et al., 2010).

Our data now show that Pmps are able to form various functional heteromeric complexes, independently of the number of motifs. As we show here, even an adhesion-inconsistent fragment of PmpA can be incorporated into a functional heteromeric complex with an adhesion-competent PmpD or PmpI. Thus, the presence of at least seven full-length Pmps and numerous proteolytically processed forms on the EB allows the formation of a very large number of homomeric and heteromeric complexes, consisting of one or more Pmp fragments. If we hypothetically consider the extreme example that all protein fragments are present, but only once in the oligomers, we would have 10^44^ possible combinations. However, the number of possibilities are likely higher. A proteomic study performed by Saka et al. (2011) showed that different Pmps are present in different amount in *C. trachomatis* EBs and RBs, suggesting that some Pmps might have a higher chance of interacting with some Pmps, rather than others. Furthermore, previous studies showed that the transcription profiles are different for each *pmp* during the infectious cycle (Tan et al., 2010) and the real abundance of Pmps in relation to each other in the *C. trachomatis* serovar investigated here is not yet known. Moreover, our data showed that heteromeric complexes formed by adhesive PmpI and PmpD did not have a synergic effect, compared to the homomeric PmpI and PmpD complexes, suggesting that heteromeric complexes might be relevant not only for adhesion, but might be also useful as a decoy for the host’s immune response. Whether the assembly of the various homo- and heteromeric Pmp complexes is regulated or occurs randomly remains to be determined. The fact that the production of the EB surface-exposed Pmp proteins is subject to high frequency on/off switching at the inclusion level by a mechanism phenotypically resembling phase variation, adds an additional level of complexity (Tan et al., 2010).

The various Pmp complexes generated are likely stabilized via disulfide bonds, as has been shown for a PmpD fragment (Paes et al., 2018). Indeed, most Pmps have multiple cysteins, suggesting that, *in vivo*, disulfide bonds play a role both in stabilizing the filamentous Pmps described here and in the interaction of the various Pmp complexes with other components of the chlamydial OM complex COMC, which is characterized by extensive crosslinking of cysteine-rich envelope proteins (Liu et al., 2010; Christensen et al., 2019). Interestingly, the functional PD of Pmps has a predicted triangular β-helical structure, suggested to be the basis for interaction (Hegemann and Moelleken, 2012). Interactions between full-length Pmps might happen *in vivo*, but it may be dependent on the proximity of these proteins on the EB surface, as full-length Pmps remain bound to the OM via their β-barrel (Henderson and Lam, 2001), and only proteolytically cleaved forms can be used for oligomer formation thus limiting filament length. This might be one of the reasons why Pmp filaments have not yet been observed *in vivo* on the EB surface. In this respect, Pmps differ fundamentally from the adhesive pili and Curli filaments, whose subunit monomers are secreted through the OM by their specific secretion machinery followed by an extracellular assembly process (Barnhart and Chapman, 2006). Instead, other adhesin proteins might offer a more realistic model for how chlamydial Pmp structures may be organized on the bacterium surface *in vivo*. For example, the cell-wall-anchored Als5p and Als1p in *Candida albicans* are characterized by the presence of amyloid nanodomains, responsible for adhesion, biofilm formation and oligomerization. In particular, Als5p forms homomeric oligomers that are displayed in patches on the cell surface (Chaffin, 2008; Chan et al., 2015; Ho et al., 2019). Similarly, FadA in *Fusobacterium nucleatum* is a secreted adhesin that forms elongated structures *in vitro*, but can also generate aggregated structures called “knots,” involved in adhesion (Temoin et al., 2012). Thus, we suggest that Pmp homomers and heteromers exists on the EB surface where they form adhesive-competent structures, possibly similar to patches and knots. So far, heteropolymeric adhesins have been described for eukaryotic pathogens only. For instance, the six PfCCp proteins of *Plasmodium falciparum* contain several adhesion domains, are differentially expressed during the parasite’s life cycle, secreted, and accessible on the macrogamete surface. Like Pmps, PfC interact with each other, assemble into multiprotein complexes that remain associated with the parasite plasma membrane, and are proposed to mediate the interaction of macrogametes during sexual reproduction of the parasite (Simon et al., 2016).

**FIGURE 7:**
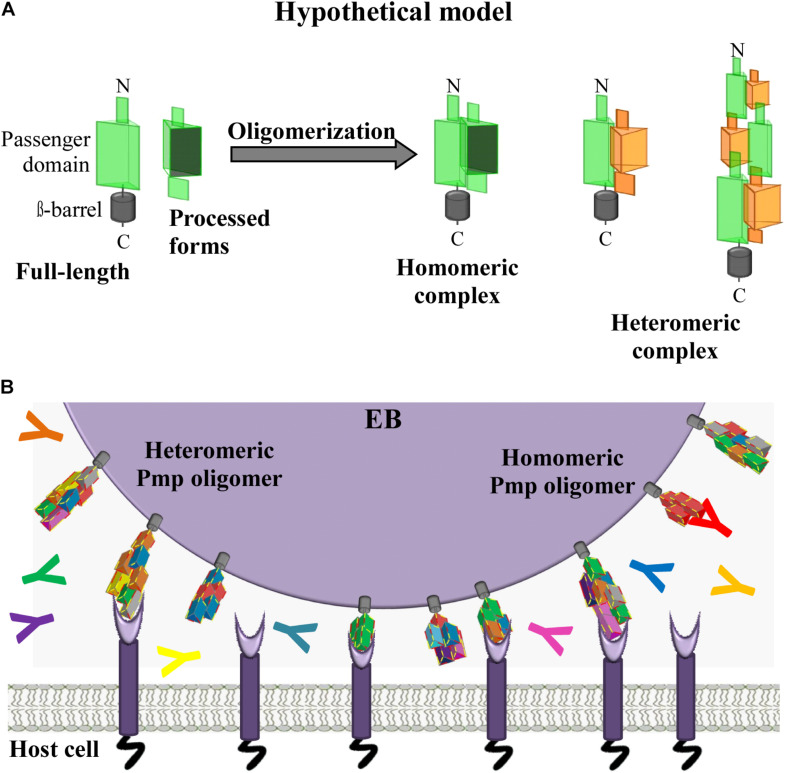
Hypothetical model. *C. trachomatis* homomeric and heteromeric Pmp oligomers are relevant for infection. **(A)** Passenger domains (PD) of the different Pmps are exposed on the surface of the EBs either in full-length or as processed forms. Full-length and processed forms of the PD of the same or of different Pmps can interact with each other, potentially forming a variety of different homomeric and heteromeric elongated oligomers adapted from Hegemann and Moelleken, 2012. **(B)** *In vivo C. trachomatis* homomeric and heteromeric Pmp structures may differ on individual infectious EBs; however, based on the adhesive capacity of their subunit Pmps, Pmp oligomers may facilitate adhesion to human cells by binding to unknown receptor(s), thus supporting EB host cell entry. Pmp-specific antibodies (coloured Y symbols) may or may not bind and block Pmp proteins, as recognition might depend on composition of the homomeric or heteromeric Pmp oligomers.

Our data provide evidence for the existence of a new class of homomeric and heteromeric autotransporter adhesin complexes, besides the known monomeric or trimeric autotransporter adhesins (Berne et al., 2015). Here, for the first time, we provide evidence that *C. trachomatis* Pmps have the ability to form adhesion-competent homomeric and heteromeric oligomers, independently of the density of motifs, which are relevant for infection. We propose that surface-exposed full-length Pmps, anchored in the OM of the EBs, interact with other full-length Pmps (of the same or a different subtype), as well as with processed PD segments derived from different Pmps, theoretically generating a very large number of different heteromeric complexes (Figure 7A). We suggest that most of the complexes formed are able to mediate adhesion to human cells. Moreover, we speculate that the potentially vast diversity of different homo- and heteromeric Pmp complexes would provide antigenic diversity and may be used by *Chlamydiae* as a decoy in the face of the host adaptive immune response (Figure 7B). Our study provides a first insight into heteromeric oligomer formation by different Pmps; however, a systematic investigation of all nine *C. trachomatis* Pmps and of their naturally existing fragments will be necessary to understand how these proteins organize themselves *in vivo*. A detailed functional characterization of the endogenous Pmp complexes during infection is a prerequisite for an in-depth understanding of the chlamydial host cell entry mechanism, as well as for the development of an efficient Pmp-based subunit vaccine.

## Data Availability Statement

The original contributions presented in the study are included in the article/Supplementary Material, further inquiries can be directed to the corresponding author/s.

## Author Contributions

AF conceived the study, performed and analyzed the experiments, and wrote the manuscript. JH conceived and coordinated the study and revised the manuscript. Both authors contributed to the article and approved the submitted version.

## Conflict of Interest

The authors declare that the research was conducted in the absence of any commercial or financial relationships that could be construed as a potential conflict of interest.
