# Supplementary material for: Chlamydia trachomatis Polymorphic Membrane Proteins (Pmps) Form Functional Homomeric and Heteromeric Oligomers
Source: Front Microbiol. 2021 Jul 19;12:709724. doi: 10.3389/fmicb.2021.709724 (PMC8326573; doi:10.3389/fmicb.2021.709724)
Supplement: Supplementary file 1 [file Data_Sheet_1.pdf]

Supplementary Figures and Table

Supplementary Figures

A

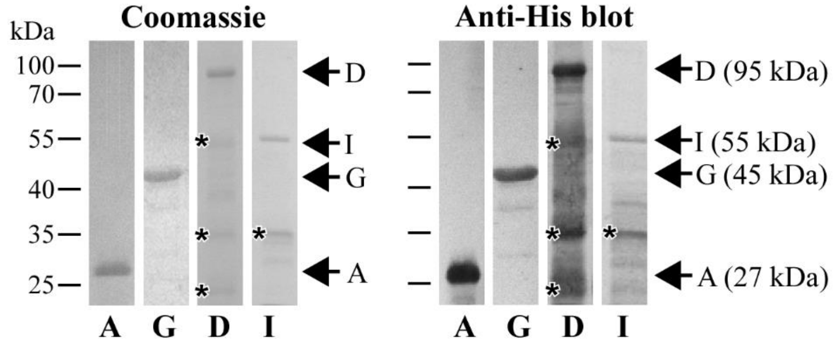

B

Pmp	FxxN	GGA(I,L,V)	Tot
**A**	2	0	2
**G**	1	1 (1,0,0)	2
**D**	18	16 (11,3,2)	34
**I**	9	6 (6,0,0)	15

Supplementary Figure 1. ***C. trachomatis*** **motif-poor and motif-rich Pmp fragments.** (**A**) Representative coomassie-stained and anti-His immunoblot of SDS-PAGE of 1 µg renatured recombinant His-tagged Pmp A, G, D and I. Arrows indicate the Pmps with their apparent MW given in brackets and asterisks indicate the main degradation bands. (**B**) Number of FxxN and GGA (I,L,V) motifs present in each Pmp fragment. Tot: number of motifs in total.

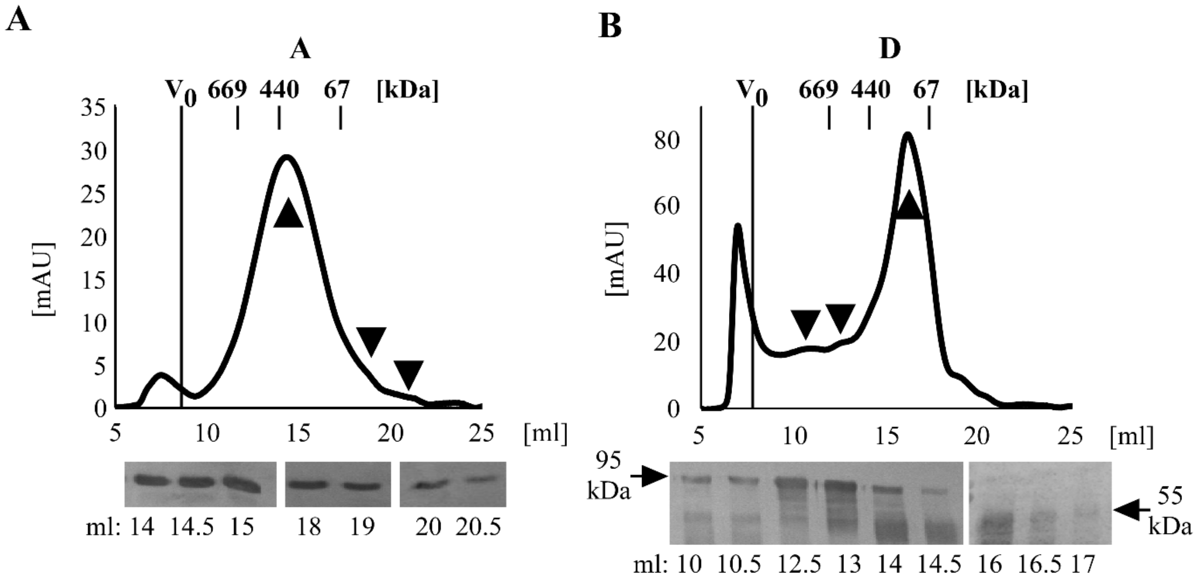

Supplementary Figure 2. *C. trachomatis* Pmps form homomeric high molecular weight (hMW) complexes. Size exclusion chromatography (SEC) curves of motif-poor PmpA (**A**) and of motif-rich PmpD (**B**), obtained with Superose6 columns. The void volume (V_0) is indicated. Elution volumes of globular standard proteins are indicated by short vertical lines. Arrowheads indicate relevant peaks. Anti-His immunoblots of relevant SEC fractions are shown at the bottom.

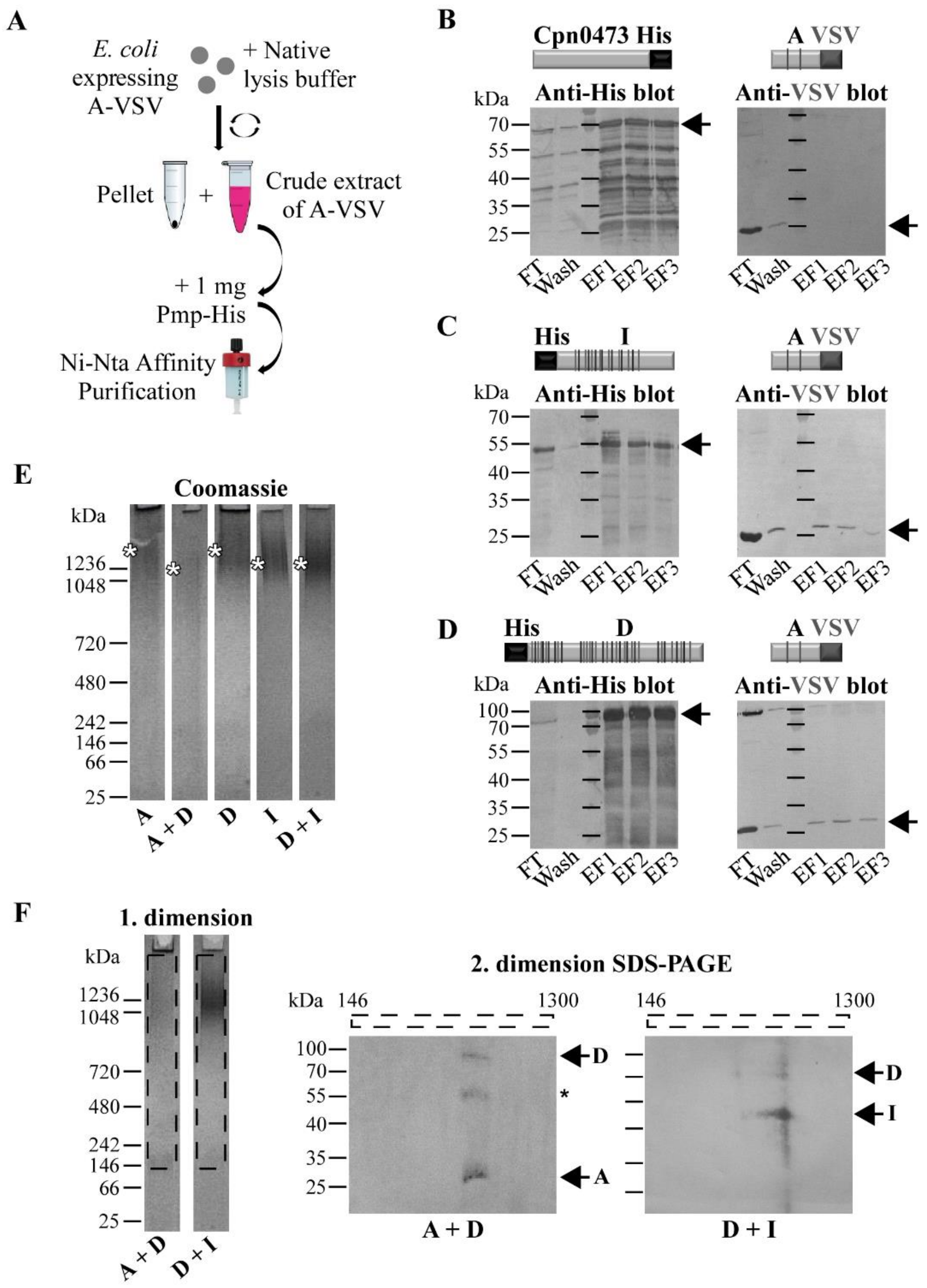

Supplementary Figure 3. ***C. trachomatis*** **Pmps form heteromeric hMW Pmp complexes.** (**A**) Outline of pull-down assay of VSV-tagged PmpA (A-VSV) with His-tagged candidate Pmps D, G and I (Pmp-His). Anti-His and anti-VSV immunoblots of pull-down assays of A-VSV with His-tagged control protein CPn0473 (**B**), with motif-rich PmpI (**C**), and motif-rich PmpD (**D**). FT: Flow Through, EF: Elution Fraction. Images are representative of one (**B**), two (**D**) and three (**C**) separate experiments. (**E**) Coomassie-stained Blue Native-PAGE (BN) loaded with 1 µg of homomeric complexes (A, D and I) and 1:1 co-refolded heteromeric complexes (A+D and D+I). Asterisks indicate the main bands. Western blots bands are cut for clarity purposes. Images are representative of three separate experiments. (**F**) Representative 1. dimension BN bands containing the hMW A+D and D+I complexes (dashed box) analyzed by 2. dimension SDS-PAGE. Arrows indicate Pmp proteins, asterisk indicates PmpD degradation band. Images are representative of three separate experiments.

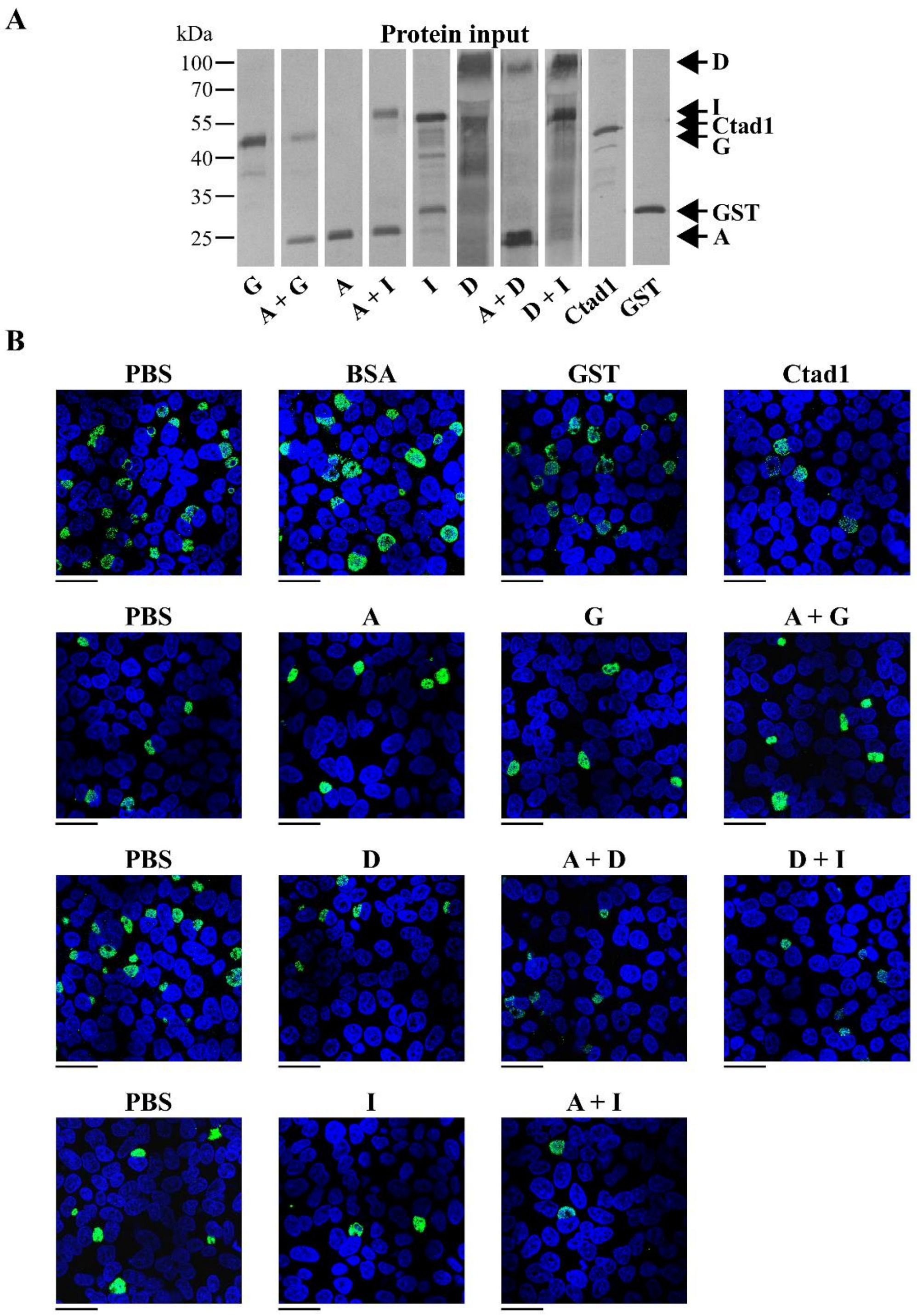

Supplementary Figure 4. Infection blocking assay with soluble homomeric or heteromeric Pmp oligomers. (**A**) Representative anti-His immunoblot of homomeric and heteromeric Pmp input samples. Arrows indicate the full-length Pmp fragments, rCtad1 and rGST. Western blots bands are cut for clarity purposes. (**B**) Representative sections of immunofluorescence microscopy pictures of epithelial HEp-2 cells infected with *C. trachomatis* at 24 hpi. Inclusions are visualized with anti-Momp antibody (green) and DNA is visualized with DAPI (blue). Scale bars: 25 µm. Images are representative of three separate experiments.

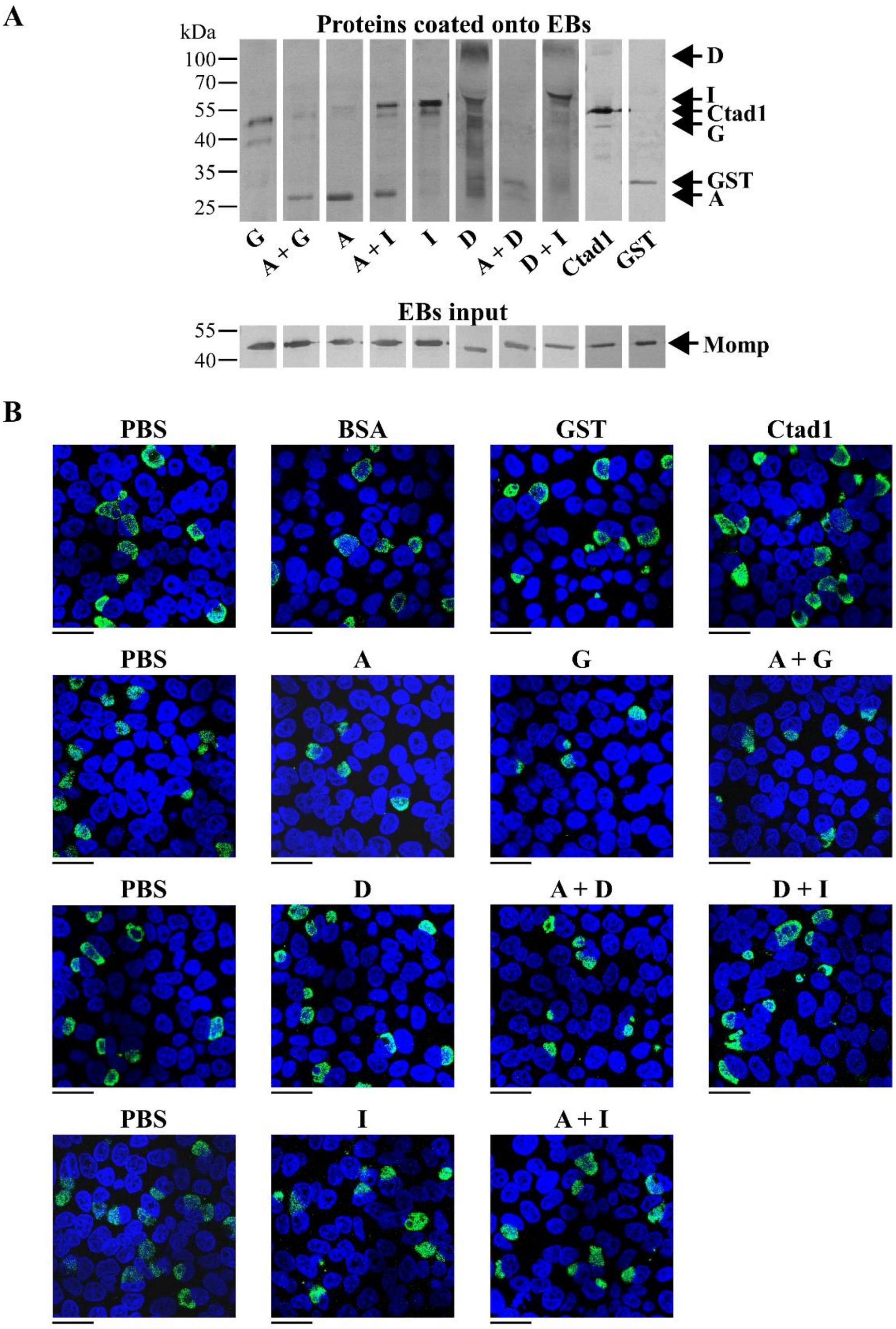

Supplementary Figure 5. Infection blocking assay with EBs pre-coated with homomeric or heteromeric Pmp oligomers. (**A**) Representative anti-His immunoblot of recombinant homomeric and heteromeric Pmp oligomers and control proteins rCtad1 and rGST coated onto EBs (top). Representative anti-Momp immunoblot of protein-coated EBs (loading control) (bottom)**.** Western blots bands are cut for clarity purposes. (**B**) Representative sections of immunofluorescence microscopy pictures of epithelial HEp-2 cells infected with *C. trachomatis* at 24 hpi. Inclusions are visualized with anti-Momp antibody (green) and DNA is visualized with DAPI (blue). Scale bars: 25 µm. Images are representative of three separate experiments.

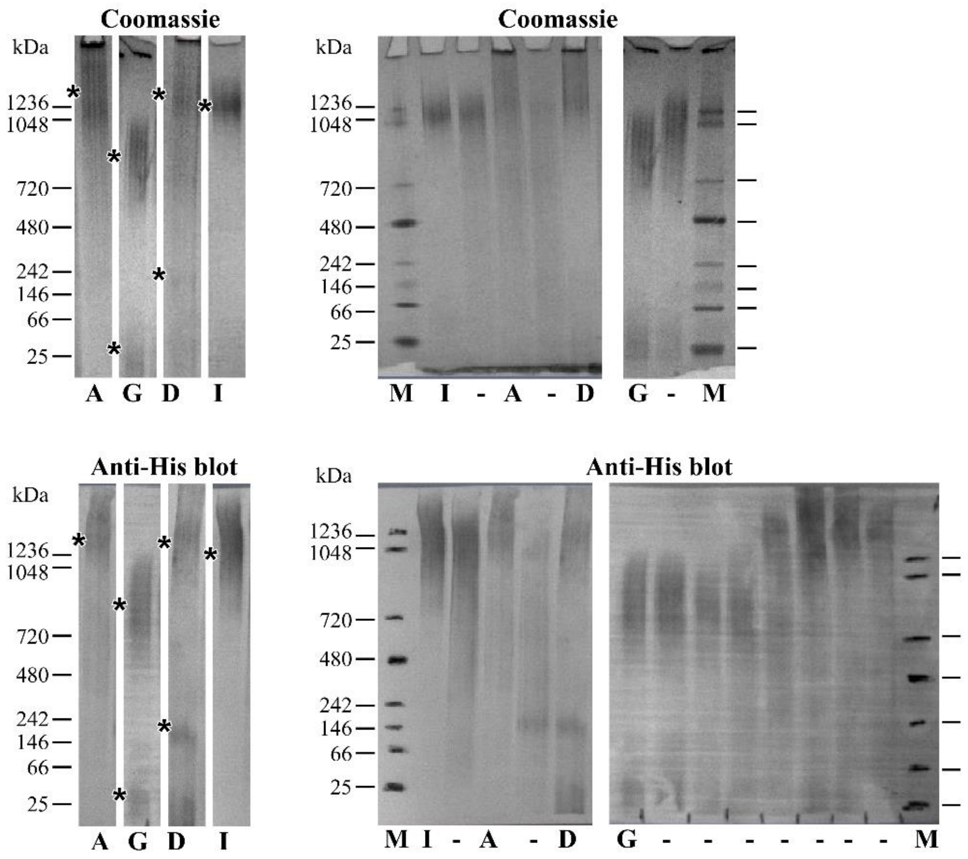

Supplementary Figure 6. Original pictures from Figure 1B. On the left, coomassie-stained and anti-His immunoblot of Blue Native-PAGE (BN) of homomeric Pmps A, G, D and I (1 µg), as shown in Figure 1B. Bands are cut for clarity purposes. On the right, original coomassie-stained and anti-His immunoblot pictures of the corresponding BN bands.

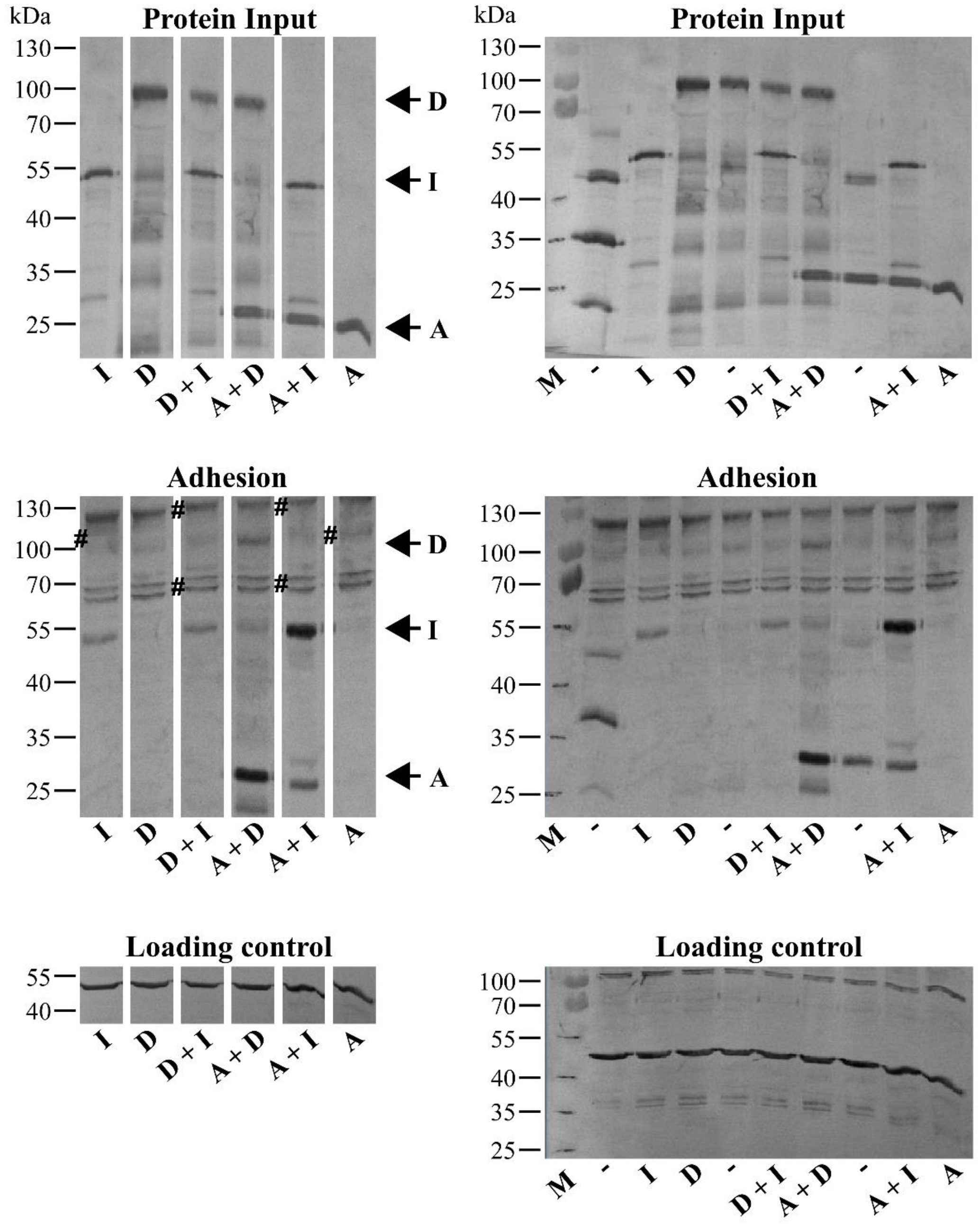

Supplementary Figure 7. Original pictures from Figure 5B. On the left, anti-His immunoblot pictures from figure 5B, representing the binding affinities for HEp-2 cells of homomeric A, D and I and heteromeric D+I, A+D and A+I Pmp oligomers (Adhesion), anti-His immunoblot pictures of input of recombinant proteins (Protein input) and anti-Actin immunoblot pictures of HEp-2 cells amounts (Loading control). Bands are cut for clarity purposes. On the right, original anti-His and anti-Actin immunoblot pictures of the corresponding western blot bands.

Supplementary Table

Supplementary Table 1. Vectors and cloning oligonucleotides for A, D, G and I *pmp* fragments.

***Pmp* fragment**	**Vector**	**Primer sequence (5´ to 3´)**
Pmp A	pET24a	AATAATTTTGTTTAACTTTAAGAAGGAGATATACATATGTCCCTAGATCGACACAATTCT
		TTTGTTAGCAGCCGGATCTCAGTGGTGGTGGTGGTGGTGGGTGAGCAAGATTTCCATTTG
	pAF14	AAATAATTTTGTTTAACTTTAAGAAGGAGATATACATATGTCCCTAGATCGACACAATTC
		GATCTCACTTTCCCAGCCTGTTCATCTCGATATCGGTGTAGGTGAGCAAGATTTCCATTT
Pmp D	pKM32	CCATCACCATCACCATACGGATCCGCATGCGAGCTCGGTATCTTTTGAAGGAAACAGCGC
		GGAGTCCAAGCTCAGCTAATTAAGCTTGGCTGCAGGTCAGAATTTCCTTTAAAAACAATA
Pmp G	pET24a	AAATAATTTTGTTTAACTTTAAGAAGGAGATATACATATGGATGGTGGAGCGATTTATTT
		TTTGTTAGCAGCCGGATCTCAGTGGTGGTGGTGGTGGTGTGCTGAATGCGCAGATCGTAT
Pmp I	pKM32	CCATCACCATCACCATACGGATCCGCATGCGAGCTCGGTAAGTTTCTGTCGAAATCATGC
		CAGGAGTCCAAGCTCAGCTAATTAAGCTTGGCTGCAGGTCGAGAGGAATATTGTTTTGCT
